# Stratification by Sex and Hormone Level When Contrasting Men and Women in Schizophrenia Trials Will Improve Personalized Treatment

**DOI:** 10.3390/jpm11090929

**Published:** 2021-09-18

**Authors:** Mary V. Seeman, Alexandre González-Rodríguez

**Affiliations:** 1 Department of Psychiatry, University of Toronto, #605 260 Heath St. W., Toronto, ON M5P 3L6, Canada; 2 Department of Mental Health, Mutua Terrassa University Hospital, University of Barcelona, 08221 Terrassa, Barcelona, Spain; alexandregonzalez@mutuaterrassa.cat

**Keywords:** schizophrenia, sex, hormones, prevalence, symptoms, treatment response, outcome

## Abstract

Background: Sex and gender differences have been reported in the prevalence, expression, treatment response, and outcome of schizophrenia, but most reports are based on relatively small samples that have not been stratified for the impact of sex hormone levels. This literature review aims to show how women’s hormone levels can impact the results of male/female comparisons. Methods: This is a narrative review of data from publications of the last decade. Results: Epidemiologic evidence, reports of the impact of hormones on cognition, results of sexually dimorphic responses to treatment, and male/female trajectories of illness over time all suggest that female hormone fluctuations exert major effects on male/female differences in schizophrenia. Conclusions: Information on hormonal status in women participants is rarely available in clinical studies in schizophrenia, which makes male/female comparisons largely uninterpretable. These are the current challenges. Opportunities for individualized treatment are growing, however, and will undoubtedly result in improved outcomes for both women and men in the future.

## 1. Introduction

Ever since 2014, when the National Institutes of Health in the United States began to require grant applicants to include sex as a biological variable in the design and subsequent analysis of all basic and animal research, sincere attempts have been made to comply. This has not been easy in studies pertaining to mental illness, because although animal models exist, these disorders are quintessentially human. Nonetheless, it is crucial to understand male/female differences in psychiatric disorders because such an understanding will improve treatment response and may offer clues to etiology.

Epidemiological data show that, in the majority of psychiatric disorders, rates of illness in men and women differ, clinical presentations differ, and treatment response differs [1,2,3]. Sexual dimorphism in the developmental course of cortical maturation results in differences in ages at onset [2]. Schizophrenia, the topic of this review, is marginally more common in men than in women, but men develop schizophrenia at an earlier age and present with more cognitive deficits, as well as an initially less robust response to antipsychotic treatment. However, after the age of female menopause, response to treatment is approximately equal. This change, plus the fact that during the postpartum and menopausal periods of women’s lives there is an increased risk for onset and exacerbation of psychotic disorders, [4] suggests that ovarian hormones influence treatment and outcome, an issue that has been relatively neglected in standard treatment guidelines. Hormone levels fluctuate throughout life [5]; testosterone has a diurnal fluctuation, so antipsychotic medications taken by men may produce somewhat different effects depending on the time of day they are taken. In their reproductive years, women’s gonadal hormones fluctuate monthly, rise during pregnancy, drop precipitously postpartum, are affected by hormonal contraceptives, drop gradually after age 40, and more fully at menopause, though they can be partially restored by hormone replacement therapy. In both sexes, hormone production can be disturbed by a variety of medical/surgical conditions, hormone blockade and replacement in transgender individuals, and in women undergoing fertility treatment [6]. There is little in the literature about the effect of hormone levels on an antipsychotic response in most of these circumstances [4], but this review will cover what is known, especially over the course of pregnancy and the transition from pre- to post-menopause in women.

A recent, extremely informative scientific statement by Bhargava and colleagues [7] addresses the many difficulties of studying sex and hormones as biological variables in pre-clinical and human studies. While both sexes produce estrogens, androgens, and progestins, brain levels of these hormones differ significantly in males and females. Moreover, they markedly fluctuate in females over time and in response to reproduction-related events. Viewing schizophrenia as a disorder of the brain, Bhargava et al. [7] make several fundamental points that will aid in the interpretation of this review: (A) While brain imaging comparisons show age- and sex-related differences in brain size, global and regional gray matter volume, and white matter connectivity, there is wide overlap, meaning that group differences do not necessarily inform either physiology or pathology at the individual level. (B) Although animal studies are important in research, rodents differ from primates in several important aspects of steroid hormone production, so hormonal results of animal studies cannot be easily generalized to humans. (C) Looking only at levels of individual hormones when making comparisons, for instance, between men and women, is misleading. While estradiol concentrations in men are similar to those in mid-cycle women, their testosterone levels are ten times higher. It is the relative ratios among hormones that count. (D) As seen in the results of animal studies, the environment (e.g., housing conditions, diet, physical activity, and time of day) profoundly affects physiology. This observation applies to at least the same degree in humans. (E) Both basic and clinical science have tended to focus on the male sex. An example of the errors this can lead to is that a drug that is found ineffective in the early stage of drug development in male rodents never makes it any further (whereas it could potentially prove to be effective in females) [8,9]. It has been hypothesized that one reason for the high rates of adverse drug reactions in women is that drugs are developed using male experimental animals and clinically trialed mostly in male humans [10].

These principles are important to keep in mind when interpreting studies of treatment response in schizophrenia. In this review, treatment response includes not only effectiveness but also tolerability and safety. The relevance here is that sexually dimorphic hormones play a large role in the occurrence and severity of drug side effects. Activities of cytochrome P450 (CYP) enzymes in the liver and gut show significant sex differences in drug metabolism in Phase I clinical trials. In addition, gastric enzymes and glucuronidating enzymes, and some efflux transporters have been shown to be more active in men than in women [11]. In addition, antipsychotic drugs are lipophilic, so sex differences in fat storage and fat metabolism affect overall treatment response [12].

## 2. Aims

Our aim is to review recent studies that have compared the prevalence, clinical presentation, and treatment outcome in men and women with schizophrenia, with an emphasis on their inclusion or omission of hormonal influences. Our goal is to question recent research on gender differences in schizophrenia with respect to the influence of sex hormones to identify current challenges and to define future opportunities for individualized treatment.

We will address the following questions:

1. Do sex hormones influence the incidence and prevalence of schizophrenia in men and women?
2. Do sex hormones influence disease presentation (age at onset, symptoms) in men and women with schizophrenia?
3. Do sex hormones influence the pharmacokinetics and pharmacodynamics of antipsychotic drugs?
4. Do sex hormones impact disease outcome in men and women diagnosed with schizophrenia?
5. Can stratification for sex hormones levels contribute to personalized prevention and treatment in schizophrenia?

## 3. Methods

We conducted a non-systematic narrative review based on electronic searches on the PubMed database for studies published in the last decade (2010–2021) that addressed our questions. The initial search strategy used the following search terms: “sex AND schizophrenia”. Our next step was to use extra sources, such as Google Scholar, to find further relevant papers in the field. Reference lists from the initially included studies were then searched to find further informative papers. We retrieved additional references at the recommendation of reviewers of the first draft of this manuscript. We apologize to the authors of any relevant studies that were unintentionally omitted.

The screening and selection process was carried out by both authors who scanned titles and abstracts, and selected papers for full-text scrutiny. We include several levels of evidence: epidemiological studies, cross-sectional studies, observational studies, and case series, but we consider randomized clinical trials as the highest level of evidence by which to reach conclusions and advance the field. Where papers refer to the same work, we cite the earlier publication. Finally, we address the potential challenges and opportunities for personalized treatment and preventive strategies.

**Figure 1:**
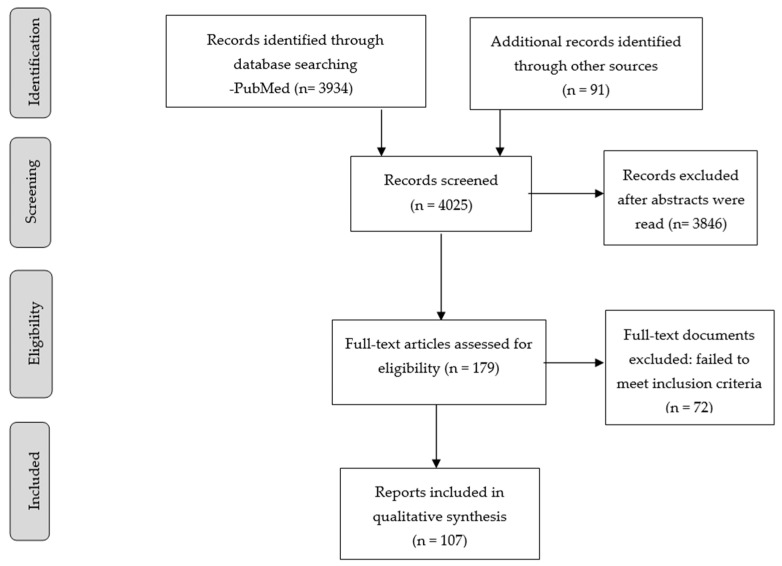
Flow diagram of included studies.

Figure 1 presents the screening and selection process, as well as the number of included studies.

## 4. Results

### 4.1. Epidemiology

#### 4.1.1. Sex Differences in Incidence

Incidence is defined as the number of new cases of a given condition in a given population over a given period of observation. The results of incidence studies are always significantly influenced by the diagnostic criteria used in the ascertainment of a given condition. With respect to schizophrenia, when the criteria are broad (i.e., when they include schizophrenia spectrum disorders), there are usually fewer sex differences reported than when the criteria are narrow [13].

The male/female incidence ratio, as reported in the comprehensive review of Jongsma et al. [13], was 1.44 for all psychotic disorders, 1.60 for all non-affective psychoses, and 1.7 for schizophrenia. There was no excess male risk for affective disorders nor for psychotic bipolar disorders, which suggests that that the influence of sex on incidence does not extend beyond non-affective psychosis.

Psychosis incidence by sex is influenced by several factors, one being onset age. In a recent study, onset over age 60 yielded an overall incidence of 0.037. With each additional onset year, the rates of women compared to men sharply increased [14]. Several investigating teams have questioned when the increased psychosis risk in older women begins [15,16]. At the junior end of the age spectrum, some have reported a female preponderance of early-onset schizophrenia-related illness prior to age 18 [17]. This is an unexpected study result and, therefore, noteworthy. It may reflect the broadness of the diagnostic umbrella utilized or it may result from this study’s inclusion of prepubescent children (prior to the influence of activational hormones). It may also represent a current increase in substance-induced psychotic disorders among young females, which serves as an important reminder of both temporal and regional variations in disease incidence [18,19].

#### 4.1.2. Sex Differences in Prevalence

The prevalence of a disorder is defined as the proportion of individuals in a population who suffer from a given condition over a given time period. Based on a well-conducted study in the first decade of this century [20], the lifetime prevalence of schizophrenia and related disorders in men and women has generally been recognized as equal. The higher mortality of men with schizophrenia due to suicide and fatal accidents has been held responsible for the discrepancy between sex incidence and sex prevalence. A second potential explanation is the inclusion of women in prevalence studies who are older (e.g., post-menopause) than women in incidence studies. The results of prevalence studies can be influenced by many other factors, such as diagnostic criteria, case ascertainment and sample selection, access to effective prevention and treatment, and environmental risk factors that affect one sex more than the other [21].

There are discrepancies in sex ratios in prevalence studies conducted in different countries; this suggests the possibility of genetic explanations. Most studies have described no sex differences in the genetic variations of risk genes. Nevertheless, recent research has reported that the Interferon Gamma Receptor 2 (IFNGR2) may act as a risk factor for the development of schizophrenia in men only [22]. The polymorphism of methylenetetrahydrofolate reductase (MTHFR) was initially shown to be a risk factor for schizophrenia more in men than in women [22]. Abnormalities in the DNA methylation/demethylation cycle and their effects on the transcriptional activity of specific genes have been reported in schizophrenia. However, in their methylation analysis, Wan and collaborators found no significant hypomethylation of genomic DNA and no sex differences [23]. On the other hand, the very recent Sershen et al., study [24] found methylation differences between men and women that were significantly influenced by antipsychotic treatment, notably by clozapine in this study.

Schizophrenia sex differences do indeed reportedly differ in different geographic populations. The lifetime prevalence of non-affective psychosis in Nigeria (varied ages) has been reported to be twice as high in men as in women [25]. This was also the case in a young population (ages 15–30) surveyed in Turkey [26]. On the other hand, a 6-year period prevalence study of 8848 patients of different ages from Israel (median age 30) found little difference between men and women [27]. A one-year prevalence study from Spain (all ages over 14) found that the rate of men was nearly double that of women [28]. A second study from Spain, this one an 8-year prevalence study of 24,749 patients with schizophrenia (ages 15–64), found a substantially higher rate for men (7.9 vs. 4.5/1000) [29]. Morgan et al. [30] from Australia found a doubled rate for men in a 1-month prevalence study. Although this study was stratified into younger and older age groups, the sex prevalence within these groups was not reported. A Thailand prevalence study of schizophrenia (ages 15–59) found the rate to be equal between men and women [31]. A very interesting result came in from a one-year prevalence survey of all non-affective psychosis in Sweden. The rate was higher in men than in women, but the sex gap decreased with increasing age until age 55. After age 55, the proportional rate reversed and became higher in women [32].

The inference from the above is that results of sex differences in schizophrenia prevalence studies, however and wherever prevalence is measured, may depend on, among other variables, the ages of the sample studied.

### 4.2. Sex Differences in Onset Age

The earlier age at onset of adult schizophrenia in males compared to females has been widely documented and is one of the classic “facts” about schizophrenia [33]. It is a wrong fact, however, because as Will Carpenter [34] states in his 2011 commentary, “It is a mistake that onset occurs in late adolescence or early adulthood. The true range is childhood to late life, and females have a different onset pattern of psychosis compared to males.” When the onset of schizophrenia is studied in cohorts under age 43, male onset is several years before that of females [35], but when the cohort is over age 43, women’s onset may well come earlier than men’s [36]. Another way of saying this is that men show a peak incidence between ages 20 and 25 whereas women are usually described as having two peaks, one between ages 25 and 30, and another after age 40.

### 4.3. Sex Differences in Symptoms

Both men and women with schizophrenia present with positive, negative, affective, and cognitive symptoms, but cognitive deficits are conspicuously more prominent in men [36,37]. Han and colleagues [38] have reported that male schizophrenia patients had more serious cognitive deficits than female patients in immediate and delayed memory, but not in indices of language, visuospatial ability, and attention. Males with schizophrenia have also been reported to show worse cognition than females on measures of social cognition, processing speed, verbal learning, and visual learning [39]. However, specific male/female cognitive results in schizophrenia remain debatable because they differ depending on the cognitive test battery, the chronicity of the sample, the presence of negative symptoms (which affects responses on tests), the age of the participants, their treatment status, and the hormonal status of the women (pre-postpubertal, pregnant, postpartum, pre-postmenopausal, menstrual stage) [40]. This latter point is important because there is evidence from animal studies that estradiol exerts a substantial effect on cognition [41,42,43,44]. Preclinical studies have shown, for instance, that mutant mice display sex-dependent schizophrenia-like behaviors in animal models [45]. Male rats in animal models show significantly more severe cognitive dysfunction than female rats [46].

The distribution of estrogen receptors in the brain aligns with subcortical structures involved in schizophrenia (the hippocampus, amygdala, thalamus, and nucleus accumbens) and with neurotransmitter pathways (dopaminergic, serotonergic, and glutamatergic) that are implicated in the neurobiology of this disorder [43]. Estrogens have demonstrated protective brain effects against excitotoxicity, oxidative stress, inflammation, and apoptosis [43]. It has been demonstrated that 17β-estradiol also regulates the expression of muscarinic acetylcholine receptors, thus directly influencing cognition [43].

In summary, although the prominence of positive, negative, and affective symptoms can also differ in men and women with schizophrenia, the effect of hormones on these symptoms is less clear than is the hormonal effect on cognitive symptoms [47,48,49].

The main findings on sex differences in symptomatic domains in schizophrenia are presented in Table 1.

### 4.4. Antipsychotic Response

#### 4.4.1. Genetic Differences

Study results of 302 Caucasian male and female schizophrenia patients aged 18–62 suggests that some catechol-O-methyltransferase (COMT) and monoamine oxidase B (MAO-B) genetic variants are associated with a sex-specific increase in the severity of negative symptoms [50]. The average age of this sample was 42 and the age range for women was between 38 and 53, so a significant proportion may have been postmenopausal, but the hormonal status was not ascertained. A study carried out by Papaleo and collaborators [51], however, indicated that functional COMT genetic variants modulate cognitive functions depending on the hormonal status of the host. This study included 229 healthy controls and 172 schizophrenia patients who were administered a cognitive assessment battery. The menopausal status of the female patients was documented [51]. Functional COMT effects were present in adult men but not in premenopausal women. After menopause, the COMT-dependent effects in adult men were also seen in women, which suggests that genetics and hormones are both relevant to sex differences in cognition in this population.

Using post-mortem tissue of elderly men and women with schizophrenia, as well as age and sex controls, sex differences have also been found in gamma aminobutyric acid (GABAergic) genes [52]. Again, if replicated, these findings may help to explain the sex difference in antipsychotic response [53].

Importantly, there are significant sex differences in the genes that code for cytochrome P450 (CYP) metabolizing enzymes [54], which determine the extent to which drugs reach their receptor targets. Significantly, partially because of genetic differences, the activity of CYPs can be differentially affected by levels of gonadal hormones. Table 2 summarizes the main sex differences in pharmacokinetics and pharmacodynamics that potentially influence antipsychotic response.

#### 4.4.2. Pharmacokinetic Differences

Sex differences in absorption, distribution, metabolism, and elimination of all medications, including antipsychotic medications are frequently discussed in the literature because men and women, on average, differ in important determinants of drug plasma levels, such as gastric acidity, intestinal motility, distribution of adipose tissue, blood volume, activity of metabolizing enzymes, and renal excretion rates [55,56]. In brief, women are more likely to absorb drugs quicker than men, and as their bodies have more adipose tissue than men, tend to accumulate lipophilic drugs such as antipsychotics [57].

These factors, plus the degree of protein binding of drugs (only the unbound fraction passes through the blood-brain barrier) and the rate of blood flow to the brain (both showing an association with sex), determine how much of the drug reaches its target(s). Most of these variables are substantially affected by age, diet, co-morbid illness, the quantity and nature of concomitant drugs, and exposure to tobacco, alcohol, or other toxic products. What is frequently not mentioned in the pharmacological literature is that they are also affected to a significant degree by the level of circulating hormones, especially estrogens [59]. Because sex hormones interact with serotonergic, noradrenergic, and dopaminergic pathways through which antipsychotic drugs are mediated [60], it is important before reaching conclusions about sex differences in the efficacy and tolerability of antipsychotic drugs, to determine the hormonal status of the women participating in a given study: have the girls reached puberty, are the women ovulating, are they pregnant or postpartum, are they on contraceptives, are they menopausal, or on hormonal replacement therapy [58,61]?

Estrogens change the activity levels of the liver and intestinal metabolic enzymes CYP1A2, CYP2C9, CYP2C19, and CYP3A4 [62]. This becomes clinically significant when treating pregnant or menopausal women, and is especially relevant to olanzapine or clozapine. Age is often used as a proxy for menopause but it is an inaccurate proxy, especially in women with schizophrenia who may not be menstruating because antipsychotic-induced hyperprolactinemia has lowered estrogen levels [57].

#### 4.4.3. Pharmacodynamics Differences

Pharmacodynamics refers to the effects of the drug on a person’s body, both the wanted effects (efficacy) and the unwanted effects (tolerability, safety, toxicity). Antipsychotic drugs block the transmission of dopamine and act on a variety of other brain neurotransmitters. Estrogen also acts on neurotransmitter receptors, sometimes to facilitate and sometimes to antagonize the effects of the drugs. In general, estrogen is thought to enhance the efficacy of antipsychotics and has been used clinically as an effective adjunctive treatment for schizophrenia [63,64,65,66]; more often, a selective estrogen receptor modulator (SERM) such as raloxifene is used [66], to avoid estrogen adverse effects. These include increased risk for breast and endometrial cancer, venous thrombosis, cerebrovascular accident, and pulmonary embolism [67].

#### 4.4.4. Sex Differences in Antipsychotic Response

How sex hormones affect drug response is easiest examined in animals. One of the most investigated rat schizophrenia models is the prepulse inhibition model (PPI) [68]. Sex-specific involvement of estrogen receptors has been found in behavioral responses to rodent stress and psychomotor activation [69], but it is difficult to apply such findings to humans.

Very few studies have investigated gender differences in antipsychotic response in humans. Ceskova and collaborators analyzed the results of the European First-Episode Schizophrenia Trial (EUFEST), an open label trial, the main focus being on gender differences in treatment response. Patients were randomized to treatment with haloperidol, amisulpride, olanzapine, quetiapine, and ziprasidone. Women treated with olanzapine (not the other drugs) had a greater improvement in PANSS total scores compared to men [70]. A population pharmacokinetic study investigating the variability of clozapine and norclozapine plasma concentrations found that women had lower clearance and thus higher concentrations of these two drugs than men and that this increased with age in both sexes [71]. A compilation study using data on amisulpride, aripiprazole, and olanzapine found higher antipsychotic concentration–dose ratios in women than in men [72]. The same data set was used to look for sex differences in response to these three antipsychotics. The results were that dosing was higher for men than for women in the aripiprazole and olanzapine groups. Dose-corrected serum levels were 71.9% higher in women than in men for amisulpride and 55.8% higher in women than in men for aripiprazole. In the amisulpride group, men responded more quickly and to a greater extent than women. The conclusion was that the influence of sex differs with the drug used, but the study included only 51 women with an average age of 33.8 and a range of 14.4 years. Many would have been postmenopausal [73], making it impossible to interpret the results.

It appears that sex steroids may influence symptomatic response to certain antipsychotics at certain ages in both men and women. The specifics are not yet known.

### 4.5. Sex Differences in Safety and Adverse Events

A person’s sex can enhance the adverse effects of antipsychotics. Because women have a higher baseline prolactin level than men, the added hyperprolactinemia induced by antipsychotics affects women more than men—it frequently leads to amenorrhea and infertility, hirsutism, acne, osteoporosis, and has been suspected of increasing the incidence of breast cancer [74]. The potential prolactin link with breast cancer remains controversial because women with schizophrenia have many other risk factors that contribute to an elevated rate of breast cancer—low parity and breastfeeding, obesity, high rate of smoking, caffeine, and substance abuse.

Hyperprolactinemia is also known to increase coagulation, thus raising the risk for venous thrombosis (VT). This may be clinically important in the schizophrenia population because adjunctive treatments such as estrogen or raloxifene themselves increase the risk for VT [75]. The VT risk to postmenopausal women on antipsychotic medication was shown in results from a large study in Taiwan. The VT rate increased in a dose-dependent fashion with age and was higher for parenteral than for oral antipsychotics. Discontinuing antipsychotics for 30 days in postmenopausal women eliminated the extra risk [75]. A gold standard for the prevention and treatment of hyperprolactinemia has not yet been established [76].

Like many drugs used in medicine, antipsychotics increase the risk for Torsade de Pointes arrhythmias. The QTc interval on the electrocardiogram is equal in boys and girls prior to puberty, but it increases after puberty in women due to the effect of estrogens on cardiac myocyte electrophysiology. Severe cardiac arrhythmia and Torsade de Pointes occur approximately twice as often in women as they do in men. A recent study investigating sex differences of various antipsychotics (olanzapine, risperidone, aripiprazole, and quetiapine) on QTc found that the mean QTc interval was longer for patients receiving olanzapine compared to those on risperidone, but that such differences were statistically significant only in women [77]. The action of testosterone and progesterone on cardiac ion channels likely contributes to this gender difference [78].

There are sex differences in the prevalence of allergies to drugs and also to excipients e.g., the non-active ingredients of pills, tablets, capsules, or intramuscular injections. Genome-wide association studies in schizophrenia have shown associations with the major histocompatibility complex and factors in acquired immunity such as B-lymphocyte lineages [79,80,81]. Thus, immune responses may be elevated in schizophrenia, with female responses differing from those of males and changing over life stages, under the influence of gonadal hormones [82,83,84].

The pattern of metabolic responses to antipsychotic drugs constitutes another important difference between men and women [85]. This is not surprising because the sexual dimorphism in lipid distribution and the mobilization of stored lipids is well known to impact tissue-specific insulin sensitivity and cardiometabolic risk [86,87,88,89,90]. Metabolic syndrome is a cluster of symptoms that includes obesity, dyslipidemias, glucose intolerance, insulin resistance, and hypertension. It is a risk factor for type 2 diabetes mellitus and cardiovascular disease and occurs significantly more frequently in patients on antipsychotics than it does in the general population. Two particular antipsychotics, clozapine and olanzapine appear to be the worst culprits [91].

Menopause has a significant effect on adipose tissue. The decline in estrogen levels promotes the redistribution of lipids in women from the thigh (subcutaneous) to the abdomen (visceral), leading to a more central adipose distribution, like that in men, which increases cardiometabolic disease risk [90].

A safety issue that is specific to women is the use of antipsychotics during pregnancy [92] and the possibility of perinatal complications for offspring. While antipsychotic medication does not seem to pose an increased risk of congenital abnormalities [93], its safety during lactation is still being investigated [94,95], as are long-term effects on child development.

### 4.6. Sex and Outcome

There are several indices of outcome used in schizophrenia research, rehospitalization rate being one. In the recent Sommer et al. study [96] on the course of schizophrenia, a 10-year follow-up of Finnish registry patients from the first diagnosis of schizophrenia or schizoaffective disorder, rehospitalization rates were found to be significantly higher in women than in men. A total of 16,148 patients were included in this study. Because the mean age of diagnosis was 38.2 in women, an unknown number of these women were probably postmenopausal. Another relevant factor in interpreting these results is that most of the patients diagnosed with schizoaffective disorder (considered a relatively mild form of schizophrenia) were women. The reasons for hospitalization are also relevant. Admission rates to the hospital because of psychosis were similar in the two sexes, as was the mean duration of hospitalization. More men were in the hospital because of substance abuse; more women were admitted because of suicide attempts, self-harm, and other psychiatric co-morbidities. This shows that the interpretation of comparisons between men and women needs to be made carefully and that, among other important variables, the hormonal status of women participants is critical [96].

Other potential schizophrenia outcome measures are homelessness, employment, marriage and parenting, substance abuse, and violence. Women with schizophrenia do better than their male peers in all those domains [97], although results depend on where and when studies are conducted. Men with schizophrenia commit severe acts of aggression more frequently than women [98], but less severe aggressive behaviors such as verbal insults and threats are more frequently associated with women. We could find no studies in the mental illness population (or in the general population) of aggression comparisons between pre- and post-menopausal women. In men with schizophrenia, testosterone levels have been shown to not be predictive of aggression [99].

Major outcome measures in this disorder are suicide and mortality rate. The lifetime mortality from suicide in schizophrenia is 4–6% [100]. Most studies report significantly higher suicide rates in male than female patients [101]. Significantly more women than men, however, attempt suicide. These sex/gender differences are similar to those seen in the general population and are understood to be multi-determined.

Worldwide, and for a number of reasons, the elderly are at a greater risk for suicide than other age groups. The number of people who die by suicide is highest in those aged 70 years or older in almost all regions of the world [102]. In Korea, one study found that para-menopausal women had substantially higher suicide rates, again for a number of potential reasons, than younger women [103]. There is no information about a differential rate of suicide attributable to menopause in women with schizophrenia.

Life expectancy is substantially reduced in schizophrenia, by over 18.7 years in men with schizophrenia and by 16 years in women [104]. The difference is probably due to the fact that men and women with this disorder experience different comorbidities. Cardiovascular death is more common in men with this diagnosis than in women [105]. Chronic smoking, however, which is prevalent among people with schizophrenia, has been reported as more of a risk for heart disease in women than in men [106], so women’s deaths may be on the rise. Men with schizophrenia are more at risk than women for substance abuse, fatal accidents, and suicide [107,108,109].

With respect to functional (educational achievement, occupational functioning, and interpersonal functioning) rather than symptomatic schizophrenia outcomes, most studies agree that women hold an advantage over men [110,111].

Recovery from schizophrenia, usually referring to a patient’s subjective sense of mastery over the illness, whether or not symptoms remain, and whether or not good functioning has been restored, have found that women are more likely than men to achieve recovery. This is true for 5–8-year follow-ups after the first episode of psychosis [112,113]. Longer-term follow-ups, however, make this conclusion far less clear [114]. Ayesa-Arriola and colleagues [115] recently reported on 1-, 3-, and 10-year follow-ups of first episode patients (95 females and 114 males). During the first 3 years, women showed a significantly superior response to treatment and significantly higher rates of recovery than men (50% vs. 30.8%). Ten years later, more women than men were judged recovered, but the difference was no longer significant (46.7% vs. 34.4%).

Recovery results appear to depend on the duration of follow-up and the age of the sample at the time of evaluation, with women initially doing better but losing that advantage in later years [97].

### 4.7. Personalized Treatment and Prevention of Schizophrenia

The circumstantial evidence strongly suggests that hormonal fluctuations, especially in women, play a role in symptom severity, response to treatment, and recovery in schizophrenia spectrum disorders. Understanding the contribution of sex and hormones may offer new avenues for personalized treatment and prevention of these disorders. Kulkarni and her collaborators [116] used the positive-and-negative-syndrome-scale (PANSS) and the Montgomery-Asberg-Depression-Rating Scale (MADRS) to determine the influence of various hormones (estradiol, progesterone, follicular stimulating hormone (FSH), luteinising hormone (LH), and dehydroepiandrosterone (DHEA)) on the psychopathology of 45 women with schizophrenia. The mean age of the sample was 46 and hormone levels were collected over 12-weeks. The investigators used group-based-trajectory-modelling of psychopathology to identify distinct subgroups. One subgroup with a lower level of symptom severity was linked to FSH, DHEA, and LH. Another group with high severity was associated with LH. The MADRS identified a depressed and non-depressed trajectory. The authors concluded that subpopulations based on psychopathology and hormone levels can be identified and that stratification of subjects into hormone-level-related groups may help schizophrenia researchers to improve existing treatments.

Hormone-dependent personalized treatment may, for women, include hormone replacement therapy (HT) at menopause. There are medical risks to HT but these are minimal if treatment is started within 10 years of menopause onset. The type of HT, its dose, route of application, and duration of use need to be individualized, taking into account factors such as age, personal health risks, time since menopause, and individual preferences [117]. Other hormone-related therapies are raloxifene adjunctive treatment for para-menopausal women, dose adjustments of antipsychotic medication over menstrual cycles, or the adjunctive use of hormonal contraceptives. Based on the effect of rising or diminishing estrogen production on their metabolizing enzymes, it may be of clinical benefit to prescribe specific antipsychotic medications and not others during pregnancy and after menopause. Lipid storage is also partially hormone-dependent, with the result that depot antipsychotics (because they are lipophilic) may not need to be given as frequently to premenopausal women as they are to men and postmenopausal women. A corollary, and important for safety reasons, is the anticipation that after discontinuation of antipsychotic treatment, men can be expected to relapse more quickly than reproductive age women. With respect to the preservation of fertility options and perhaps, too, to prevent breast cancer, osteoporosis, and post-menopausal venous thrombosis, it would be safest to treat women with prolactin-sparing antipsychotics. A potential form of schizophrenia prevention in girls showing early signs of psychosis risk or a strong family history of schizophrenia is to accelerate puberty by extra feeding [118] because early increased estrogen level has been shown to delay the onset of symptoms [119]. These possibilities should be investigated in animal models of schizophrenia.

## 5. Discussion

It seems clear that schizophrenia research that aggregates data from combined groups of men and women can obscure clinically meaningful data. For instance, when studying antipsychotic dose effects, it is not sufficient to tailor the dose to body weight or body surface. It is important to also take into account effects such as lipid distribution and sex-specific pharmacokinetics and pharmacodynamics in order to arrive at optimal effective and safe drug doses for men and women.

Gene expression, cellular regulatory pathways, and many physiological functions differ between females and males. The main point of this review is that hormones, endogenous and exogenous, play important direct and indirect roles in treatment response [120].

Currently, there is limited literature evaluating the question of how circulating levels of sex steroids impact drug efficacy or adverse reactions. There exists no sex-specific (nor pregnancy or menopause specific) labeling or dosing recommendation. This will be needed as psychiatry moves toward individualized treatment.

Psychiatric textbooks and classroom teachings tend to describe psychosis as it is experienced by men. There is a feminine side to psychosis that does not make its way into the psychiatric literature nor the education of psychiatric trainees. Women tend, for instance, to experience psychotic symptoms as a result of triggers that are different from those of men. In women, these triggers are often hormonal. The timing of onset of diagnosable illness in women is considered late by male standards, and the discontinuities in the course of illness (many well periods interspersed with psychotic episodes) are more characteristic of women than of men, perhaps due to hormone fluctuation. So are the association of flare-ups with immune and hormonal changes, and the relative responsiveness to treatment [121].

## 6. Conclusions

In addressing the 5 questions outlined in our original aims, we conclude that there is evidence that sex hormones, at least estrogens, are protective against schizophrenia—they contribute to a decreased incidence of this disorder in women, to delayed onset of symptoms in young women, to a second onset peak in women in the years preceding menopause; estrogen loss is probably responsible for the increased prevalence in women relative to men after middle age. Although the presentation of schizophrenia differs in women and men (in general more affective symptoms in women, better preserved function, more negative and cognitive symptoms in men), these differences are not easily tied to hormones, the exception being that estrogens are known to protect against cognitive deficits. With respect to outcome, many prognostic facets seem to favor women, but these can be strongly influenced by gender-related variables other than hormone levels. There is evidence that female sex hormones improve the response to specific antipsychotic drugs and that the addition of estrogen or SERMs to antipsychotic regimens results in benefits. On the other hand, hormone-influenced metabolism of at least some of these drugs differs in men and women and contributes to, on average, more adverse effects in women.

Insufficient attention has been paid in the laboratory and the clinic to optimizing treatment needs for women with schizophrenia. The ‘one size fits all’ concept of drug development and clinical dosing guidelines is not appropriate for personalized medicine. Some journals and funding agencies are adopting policies to promote sex analyses in drug development, but probably not quickly enough [62]. In human clinical trials, it is important to investigate and aggregate data according to not only sex but also hormonal status, at least in women. In addition, sex-specific information needs to be made easily available to prescribers and patients on drug websites and prescription labels. The literature reviewed here suggests that this will significantly improve the effectiveness of schizophrenia spectrum disorder treatment.

## Figures and Tables

**Table 1:** Sex differences in psychopathological and cognitive symptoms in schizophrenia.

Animal Models	
Global findings	Ovariectomized rats show low levels of estrogens with hyperfunction of the dopaminergic system (an animal model of menopausal psychosis) [41,45].
Findings by hormonal status	17-betaestradiol combined with antipsychotics reversed amphetamine-induced latent inhibition disruption in rats [46].
**Human Models**	
Global findings	Men with schizophrenia show worse immediate and delayed memory and worse social cognition, processing speed, and verbal and visual learning than women [37,43]. The proportion of psychopathological symptoms may differ by sex but their variety shows no sex difference [43].
Findings by hormonal status	Estradiol has a positive effect on psychopathological and cognitive symptoms [43].

**Table 2:** Sex differences in antipsychotic response.

Pharmacokinetic Differences	
Absorption [55,56]	Women absorb antipsychotic drugs better than men during their reproductive years.
Distribution [57]	Women’s bodies have more adipose tissue within which antipsychotic drugs can accumulate.
Metabolism [57]	Estrogen levels impact some, but not all liver and intestinal metabolic enzymes.
Elimination [57]	Hepatic clearance is lower in premenopausal women than in postmenopausal women or men.
**Pharmacodynamic Differences**	
Efficacy (wanted effects) [57,58]	Premenopausal women show a better response to antipsychotics compared to men and postmenopausal women.
Side-effects, safety concerns (unwanted effects) [58]	Women suffer more frequently than men from cardiac arrhythmia, obesity, the effects of hyperprolactinemia, immune reactions.

## Data Availability

Not applicable.
